# Queering the Social Imaginaries of the Dead

**DOI:** 10.1080/08164649.2020.1791690

**Published:** 2020-07-23

**Authors:** Margrit Shildrick

**Affiliations:** Department of Ethnology, History of Religions and Gender Studies, Stockholm University, Stockholm, Sweden

**Keywords:** Death, social imaginaries, hauntology, temporality, Ireland

## Abstract

I offer a philosophical examination and feminist queering of the social imaginaries of the dead – with specific reference to recent public disclosures about death in Ireland’s Mother and Baby Homes – by looking at the issue of spectrality through the work of Jacques Derrida and others. What does it mean to respond to the dead, who, though temporarily forgotten, return to haunt us not as remembered human beings but as remnants or remainders? The normative distinctions between past and present; past, present and future; between living and non-living; absence and presence; and self and other are all made indistinct when displaced by a non-linear temporality. What differential is in play with respect to those who are grievable (in Judith Butler’s terms) and the others who constitute what Giorgio Agamben calls bare life? The strategy of memorialising the re/discovered dead seems inadequate, and I outline an alternative hauntological ethics, as suggested by Derrida, and ask if there are queer social imaginaries that allow us to live well with the dead not because we give respect, but because death itself has been rethought. I close with some speculations arising from Deleuzian vitalism and Rosi Braidotti’s optimistic claim that ‘death frees us into life’.

What does it mean to respond to the dead, who, though temporarily forgotten, return to haunt us, not as remembered human beings but as remnants or remainders? The normative distinctions between past and present; past, present and future; between living and non-living; absence and presence; and self and other are all made indistinct when death refuses to settle. The time is out of joint, displaced by a queer non-linear temporality. The use here of the term ‘queer’ indicates a thorough-going critique of normative thought that emerges both as a response to particular conditions of precarity and as a way of opening up a different understanding of futurity. It is a mode not so much of conscious resistance, as one of suspension, a portal perhaps to an ontological shift. My aim is to offer a philosophical and feminist examination and queering of the social imaginaries of the dead, with specific reference to the recent public revulsion invoked in the face of disclosures about the multiple deaths in Irish Mother and Baby Homes which flourished throughout the twentieth century. Although Ireland is my focus, there are resonances with related issues in many other places where the colonial matrix of power has operated, as well as those societies that have institutionalised and othered groups who have failed to meet normative standards: the deaths of indigenous children in the notorious Residential Schools of Canada, Russia, the United States and Australia are major sites of the former,^1^ while the notorious Aktion T4 program in Nazi Germany, and the countless lost lives of those incarcerated in multiple countries by systems of ‘care’ for developmentally disabled adults and children exemplify the latter.

Following the re/discovery – for they never went away – of those dead lost to public discourse in Ireland and elsewhere, what has been evoked is a new sense of civic obligation to recover their ghostly voices and stories, and then to reinter them so that they (or rather we) may rest in peace. I will suggest first that although such a strategy of memorialisation is an important response to familial bereavement and social outrage, it may also re-enact the original offence and fail on the grounds of both responsibility and justice. I will start by laying out the empirical context, albeit with some ethical and political observations, before moving forward in another register. My suggestion is that there are social imaginaries that allow us to live well with the dead, not because respect is demanded and given, but because the life/death binary has been rethought. What will be outlined, then, is an alternative hauntological ethics, as suggested by Derrida, and rather than perpetuating the brute binary of living and non-living (with a dash), my use of the term ‘non/living’ with a slash^2^ indicates a state not of inertness or of the inorganic but of, at very least, spectral presence. What differential is in play with respect to those who are grievable in Judith Butler’s sense, and the others who seemingly constitute bare life? Whilst exploring a characteristically Derridean analytic of spectrality as my primary point of departure, I will ask how we might learn to live well with the dead, and I will close with some brief speculations on a further alternative promise arising from Deleuze’s understanding of vitalism and Braidotti’s optimistic claim that ‘death frees us into life’ (2006, 211).

The immediate problematic of living well with the dead opens, then, on to some of the recurring scandals that have shaken social imaginaries in Ireland and challenged the image and authority of the Roman Catholic Church throughout the world. What has been publicly emerging in recent years has been a confrontation with the consequences of the Church’s policy – in collusion with the Irish State – of cleaning up the putatively shameful spectacle of illegitimate births. That alone demands a feminist response to the sociopolitical oppression of vulnerable women, and it is important to note, although I will not develop it here, that the uncared-for dead in this instance mark the exercise of patriarchal power. The greater concern of this article is that recent and ongoing disclosures about Ireland’s Mother and Baby Homes speak to the question of how the non/living disturb temporality, ethics and ontology alike. As Derrida puts it ‘A specter is both visible and invisible, both phenomenal and nonphenomenal: a trace that marks the present with its absence in advance’ (Derrida and Stiegler 2002, 117). And he goes on to call for a hauntological ethics that might figure a new imaginary of living well with the dead. Derrida is not often directly associated with queer theory but his contestation of conventional norms and sociopolitical values suggests precisely such a reading, as Carla Freccero’s take up of hauntology in her book *Queer/Early/Modern* (2006) powerfully suggests. She describes that text as

a work of mourning and thus a working through … [of] queer spectrality as a phantasmic relation to historicity that could account for the affective force of the past in the present, of a desire issuing from another time and placing a demand on the present in the form of an ethical imperative. (Dinshaw et al. 2007)

In that respect, and going well beyond the concerns of sexuality (though the issue of women’s supposedly aberrant sexuality saturates the specific Irish context), queer theory – as Mel Chen (2011, 278) claims – has always been underpinned by matters of life and death. However much relations with and of the non/living are managed and contained, there is always something queerly excessive and unruly.

Before turning to that theoretical perspective, let me review some of the empirical issues that have forced themselves into public awareness. In 2017 in County Galway, Ireland, a tenacious local historian, Catherine Corless, began to uncover the story of what had taken place in the last century at the Tuam residential home run by the *Bon Secours* order for what would no doubt have been described at the time as wayward and sinful women. Most of those admitted to the facility were pregnant but unmarried, although it appears that others were simply deemed to be in moral danger, or had been sexually abused, often by close relations. It figures the familiar story of patriarchal control over women’s sexuality, though in this case – and one fears many others – with the consequence that innumerable lives were ended before they had hardly begun. Certainly the young women themselves would have been considered and treated as worthless, but what has appalled public sentiment since the story broke has been the sheer numbers of neonates and infants who were allowed to die. It appears that between 1925 and 1961, almost 800 such children were buried en-masse near to the convent.

The original media story that the bodies were discovered in a septic tank is sensationalist, although, following forensic excavation, recent official updates do indicate that it may indeed have been some kind of sewage site. What has particularly dismayed the Irish public is that an initial report of unexplained human bones being found by some children at play in the locality of the *Bon Secours* home was never followed through by any religious or civic body. More recently, the Irish – and subsequently world – media enthusiastically took up the story both as an horrific narrative of specific neglect, but also as another assault on the integrity of the Roman Catholic Church in an era in which the uncovering of sacerdotal abuse has become widespread. The claim that nobody knew is, of course, unsustainable; plenty of people knew and chose not to act until Catherine Corless forced the issue. The exposure of the punitive conditions imposed on young women effectively incarcerated by the Church to work as unpaid labour in laundries, and the wholesale trafficking of young children born illegitimately for adoption by those who could handsomely remunerate the church, was already in the public eye through such films as *The Magdalene Sisters* (2002) and more recently *Philomena* (2013). The surprise – if there is any – is that what was surely widely known has for so long remained beyond discourse.

Undoubtedly the sheer numbers of deaths at Tuam is shocking, but what seems to have caused as much concern was that the babies and young children were disposed of in unmarked sites, not even a mass grave – where that implies some kind of recognised burial ceremony. The public expression of shock is itself somewhat curious in that Ireland – and particularly in rural areas – has a long history of burials in *cillíní.* These are similarly unmarked, unconsecrated, and unspoken of sites where unbaptised babies were semi-secretly buried alongside a variety of putative wrong-doers. The specific discoveries at Tuam are, then, proceeded by another disavowed context. The *Bon Secours* nuns running the facility did issue some death certificates, but there is no documentation of date or place of burial, and it may yet turn out that the numbers of *recorded* dead are unreliable in being deliberately inflated to cover over a baby farming operation. Whatever the absolute numbers turn out to be, there was, then, no naming of the dead at the time, still less any memorial to them; simply a stripping away of identity that reinforced the illegitimate children’s short-lived status as ‘bare life’ to use Agamben’s (2008) term. Neither the babies nor the several young mothers who died in childbirth were considered grievable. That pattern has so often been repeated elsewhere: the dead children of the Indigenous Residential Schools were often buried without any record of names, gender, or cause of death; while, to take just one specific example, at the Huronia Regional Centre for developmentally disabled children in Ontario,^3^ the vast majority of graves were either unmarked or simply assigned a sequential number. That site was considered to be of such little importance that a large sewage pipe was subsequently run through it.

In Ireland, the policy of deliberately dehumanising the unwanted extended too to many young women ‘taken in’ by Catholic orders and often forced to assume new names imposed by the nuns who supposedly cared for them. In popular culture, the resonances throughout with Margaret Atwood’s (1998 [1985]) monitory story *The Handmaid’s Tale* are all too clear,^4^ while most of us will be aware of the very same processes as a tool of colonial expansion into indigenous lands, and as a ubiquitous facet of the slave trade. Perhaps it is the fear not just of being unknown but of being deliberately un-named, of having one’s identity denied, that has most resonated in what is a uniformly disturbing history. The imposition of modernist hierarchies of value that such a tactic of epistemic and ontological violence enacts is a now familiar aspect of the analytic developed by decolonial theory, which resonates strongly with the Irish situation. Death has not always been literal; the violence of cultural erasure, however and wherever it is manifest, performs a similar function.

What is in play here clearly evokes Judith Butler’s exposition of grievability:

the differential allocation of grievability that decides what kind of subject is and must be grieved, and which kind of subject must not, operates to produce and maintain certain exclusionary conceptions of who is nominally human, what counts as a liveable life and a grievable death. (2004, xiv–xv)

Her analysis is explicitly linked to the event of 9/11 and the subsequent war in Afghanistan, but the dehumanisation of those who died at the hands of U.S. military force is just as cogent in the context of the public effacement of the women and children ‘othered’ by both Church and State in twentieth century Ireland, and in the devaluation of indigenous populations or people with disabilities. As Butler sees it, the ‘Other’ is derealised, afforded the status of neither the living nor the dead, but left, we might say, in a state of limbo. This both chimes with the long-standing Roman Catholic doxa that unbaptised babies are assigned to Limbo,^5^ and curiously aligns to Derrida’s notion of spectrality, of which more later. In Butler’s own terms those in a state of suspension between life and death cannot evoke loss, or appeal to the commonailty of a shared vulnerability. In other words, ‘there will be no public act of grieving’ (2004, 36).

The events at Tuam are now central to the belated call for responsibility and justice, even though that specific scandal was preceded by reports as far back as the early 1990s of very similar circumstances at multiple other sites, including Castlepollard, Sean Ross Abbey in Rosecrea, Pelletstown in Dublin and in a suburb of Cork, the Bessborough Mother and Baby Home, run during the period in question by the *Sisters of the Sacred Heart*, where unmarked burials took place as late as 1990. All are now among the 14 sites that are to be investigated by the *Mother and Baby Homes Commission*, though it is hard to imagine that that is anything more than a token number. In the space of just 19 years in the mid-twentieth century, almost 500 infants and a handful of adult women were recorded as dying at Bessborough and were buried in a number of unmarked mass graves at various sites.^6^ It is worth noting that unlike the absence of record keeping in Tuam, the nuns themselves reported the high numbers of deaths, again raising the possibility that it was to cover over the fact that the children were actually put up for illegal adoption, often to wealthy American families able to generously remunerate the Church. At the time of the first public disclosure of the Bessborough scandal – whatever it entailed – a lengthy report was submitted to the *Irish Health and Safety Executive* in 2012 but that text has never been published. The cover up was hardly surprising insofar as the document notes that ‘the records themselves expose a telling indictment of what may have been one of Ireland’s most damning and destructive partnerships of collusion, corruption, and abuse between Church and State’ (ÓFátharta 2015). What became clear was that a doctor inspecting the Bessborough home during the 1940s had already expressed her dismay to the authorities at the high number of declared deaths and the desperate lack of appropriate health and emergency care given to the women and their babies.^7^ She records an extraordinary level of neglect and a punitive refusal of medical services on the grounds that the supposedly errant women *should* be made to suffer. There have been – as with Tuam since 2017 – a number of press reports about Bessborough since 2012 but never the same scale of public recognition or disquiet, and until quite recently, no significant calls for justice. How, then, could the difference in response be explained? What makes one set of circumstances so disturbing in its silencing of the dead that it initiates a state *Commission of Investigation*,^8^ while the broadly contemporaneous events at Bessborough were in a sense reburied until Tuam let loose the spectres again? Could it be to do with naming? I’ll come back to this when I outline Derrida’s proposal of hospitality to those spectral others who are dead but still address us – those I am calling the non/living. Like many others, I am myself an avid reader of gravestones and realise at some intuitive level that seeing a name and a date has a very settling effect. In the cathedral nearest to my home, there are several huge gravestone slabs recording the multitudinous deaths of young children from Liverpool Orphan Asylum with a noticeable spike over a 3 year period during the early 1870s. I doubt those orphans had much effective medical care either, and though one feels sadness at the circumstances of their demise, they do not disturb me. Each has a very precise name, age and date of birth. The gravestones convey a gesture at least towards propriety, in the sense of what is one’s own. The orphans will never be spectres that haunt in any negative sense.

In stark contrast the Tuam, and to a lesser extent the Bessborough bodies are unknown others out of time, and what public sentiment demands – and I read it as a term of identity – is a proper burial. In commentaries around the events, the call for reinterment and the recognition that that entails is strong and persistent. It could be understood as an undoing of the categorisation of the unfortunate women and babies as bare life; that they were not after all nameless animals stripped of all dignity and entitlement to rites. I am reminded too of the wrenching list, recently published in full by many newspapers in Europe and others worldwide, of all the 34,361 migrants and refugees known have died in the last 25 years fleeing violence and oppression and attempting to found new lives within the European Union (UNITED 2018). For all the efforts to give the deceased some recognition, so few of them are personally identified. The unnamed and uncared for bodies of the dead may always appear as especially defenceless and vulnerable such that giving them recognition is a step towards righting the wrongs. In another context that same urge is equally evident in Canadian groups like *Remember Every Name* who seek to memorialise the dead of the Huronia Regional Centre. There is a clear sense in which naming the person is strongly aligned with naming the injustice done to them. But the dead – those dead and these dead – still have the power to haunt, so I wonder, too, if we can live well with the dead only if they are kept in place by the process of naming, only if they become objects of knowledge? It chimes with an almost overwhelming temptation to domesticate what strikes us as challengingly anomalous, to give a name not for the sake of the other but for ourselves. In Catholic theology, naming the dead *is* about remembrance, but we cannot ignore the subtext that it also facilitates a settling and forgetting.

There is so much more that one could, and perhaps should, say about the empirical basis of the shock generated by Tuam and Bessborough and other similar scenarios. I say ‘similar’ very deliberately to avoid the implication that there are one or two exceptional moments for which specific people or institutions alone can be held responsible. Like the inhumanity perpetrated against women in the Magdalene laundries,^9^ what happened at Tuam and Bessborough is surely replicated across faiths, nationalities, and historical time. I am well aware that in equivalent Protestant run homes in many other countries, unwed and supposedly unworthy mothers were routinely incarcerated and encouraged – or forced – to hand over their infants. One must think too of the power of colonialist endeavours worldwide to reduce whole populations to bare life, and render them disposable, as the English Ascendency undoubtedly did to the Irish themselves, or the Euro-Canadian and Australian authorities to indigenous peoples. Whatever the register, it exemplifies the violence that sets out the limits of Euro-American modernity. The underlying histories of oppression, and the resistance – or at least some forms of resistance – to them figure an intersectionality that might be thought most effectively through the mode of decoloniality. It is not just the recognition that Ireland was colonised and that the subsequent state and religious hierarchy adopted and expanded on existing institutional practices after the advent of the Free State (O’Donnell 2018), and went on to enshrine harsh gender discrimination against all women in the 1937 Constitution. It is that more contemporary State and Roman Catholic strictures over the reproductive lives of women have generated a counter decolonial way of thinking that restores consequence to those marked as bare life.

Following Foucault we have come to think of modernist biopolitics as circulating around ‘the power to *make* live and *let* die’ (2003, 40) which shades swiftly in a kind of thanatopolitics where bare life (Agamben 2008) – the category of *zoë* – denotes that which is inscribed with death from its conception. Death may be passively allowed more than actively pursued, as it had been under sovereign power, but it is just as implacable. I would strongly suggest that the events at the Mother and Baby Homes, and analogous sites, exemplify such a thanatopolitics, particularly in its distinctive overdetermination in relation to those of limited economic worth. The issues of gender, ethnicity and class are at the forefront. It is in such a context that the Pope’s 2018 visit to Ireland proved so inadequate. Though he sought to atone for the widespread sexual abuse of children by those in holy orders, he failed to acknowledge, or perhaps even recognise, other wrongs done, not least to women and children in Mother and Baby Homes. His expression of the Church’s shame and plea for forgiveness failed to satisfy most activists who gathered at *Stand for Truth* events with placards demanding TRUTH, JUSTICE, LOVE. But what such a truth could mean, or how it could be definitively established remains unclear.

Even the specifically Christian idea of shaming, which finds its counterpoint in many other religions, is no straightforward notion. That it is so often called *abject* shame signals precisely the desire to provide a scapegoat that distances the right-thinking majority from the errant others who were once our own: not us, not here, not now. Initially of course it was the wayward women who were shamed, those who were marked as bare life and whose infants, at the time, were scarcely grievable. The question of who knew is usually assumed to be central to the delivery of justice, but like the disputed culpability of the ordinary Germans under National Socialism who turned away from the death camps, and the T4 program that sanctioned the killing of disabled infants and children, and later adults, it is rarely self-evident. The desire to find those who are guilty – a secondary abjection if you like – is already complex at every empirical level, though I do not want to suggest that the final impossibility of full justice as such (and I follow Derrida (1992) in this) should cover over either the aspiration or its incomplete fulfilment. What is necessary is to move beyond the idea that historical wrongs can be brought to light and through a process of censure, shame and recompense be effectively reburied. That cannot be what living well with the dead entails.

The cause and effect notion of temporality that a conventionally modulated history suggests – the ‘facts’ and their consequences that I have already referred to – is in any case hard to sustain in any radical understanding of how history is constituted. In this age of post-truth politics, it is perhaps only too clear how shaky are the foundations for any belief in what ‘really’ happened. Is the power that demands a reckoning for new discoveries any more acceptable than the power (which I have polemically called patriarchal and colonial) that initiated oppressive events and then covered them over? The significance of the spectral dead lies not in explanatory accounts of materialised phenomena, but in the discursive production of those accounts, not just in terms of ideological interests, but as a matter of psychic investments in prevailing social imaginaries. The repressions, fears and desires that underlie the literal and metaphorical unearthing of bodies should forestall a merely descriptive reading of the historical sources. In approaching the material in this way, I want to signal an unwillingness to limit the significance of historical and contemporary accounts to their constative content. As Derrida and others indicate, the statement ‘x happened’ is better understood in the form ‘x will have happened’, meaning that the past is still before us, awaiting interpretation. And should archival sources exist – the death certificates, say – then we cannot unproblematically privilege them. As Dominick LaCapra warns:

The archive … is a literal substitute for the ‘reality’ of the past which is ‘always already’ lost for the historian. When it is fetishized, the archive is more than the repository of traces of the past which may be used in inferential reconstruction. It is a stand-in for the past that brings the mystified experience of the thing itself. (1985, 92)

In a telling pun, Derrida refers to archives as hierarchives, and in one of his last interviews he reminds us of the need for vigilance around what we think we know of the past. As he remarks: ‘every politics of memory … implies the intervention of a state’ (2007, 62).

Of course we do make allowances for the original context of any record – the religious and social hostility to unregulated sexuality in mid-twentieth century Ireland that silenced the disregarded 1940s report of the Bessborough physician, for example, or the long history of *cillíní* – but that is only a starting point. It is not simply a matter of acknowledging the processes of selection, suppression, and narrative manipulation that constitute any archive, but of questioning the adequacy of analyses which focus solely on conscious or intentional motivations. The promised report from the Irish state’s *Commission of Enquiry* will be, I suggest, both a matter of authoritative command – an ideological incitement to ‘Believe this!’ – and a question of subconscious desires and fears. As Derrida puts it: ‘It is also necessary to mobilise psychoanalysis with respect to … the work of mourning, collective memory … spectral traces of all kinds’ (2007, 137). In other words, the complexities of meaning are not limited even to a recovery of the implicit as well as explicit content, but are fully imbricated with our own present, and future, context.

The status of an historical account, then, is never straightforward, and rather than faithfully reproducing some putative original, it is a discursive product, constituted both in recovered materials and in the subsequent reiteration of those materials. The pressure is to deliver clear narratives, to give a voice to bereaved mothers and other witnesses or survivors, to respect their accounts but not to acknowledge that they and we are agents in a projective enterprise. What is at stake for a postconventional approach is not, of course, a matter of truth or falsehood, but rather the production of meaning through a process of reiteration that both reinforces the supposed ‘veracity’ of the event, and simultaneously destabilises it. Again as Derrida (1988) makes clear, reiteration is never simple repetition, but always introduces slippage that belies the fixity of any event such that the meaning of history is always in the future (Derrida 1996, 36). This suggests a radical departure from the notion of temporality as a succession of ‘now’ moments that confidently assert the distinct realities of past, present and future. It is not a teleological progression but one of discontinuities, loops and emergence that radically destabilise normative expectations.^10^ This is strongly mirrored in queer theory which is equally marked by indeterminacy, provisionality, openness, and a sense of the *avenir* – the figure of Derrida’s monstrous *arrivant* – that which has not yet come, be it from a past or from a future (O’Rourke 2005). The further significance of such a rethinking is, as Tamsin Lorraine notes, that: ‘the reconception of time is a crucial component of a “post-modern” ethics’ (2003, 31), which situates responsibility in the refusal of any final interpretation. For all the seeming abstraction, there is a substantive context.

In my analysis of the concept of living well with the dead, I want, then, to resist closure of meaning and urge an approach that is deliberately open-ended and undecidable. As Kelly Oliver writes in *Witnessing*, the task is not to recognise ‘the familiar to confirm what we already know’, but to listen ‘for the unfamiliar that disrupts what we already know’ (2001, 2). The fissures, breaks and contradictions in the newly exposed history are not then problems to be resolved, but opportunities to configure an ethics, not of belated recognition, but of response and responsibility. It needs to be stressed that however we conceive the dead, our encounter with them must always engage with some form of ethics. As we progressively connect with the dead of the Mother and Baby homes, it is imperative not to turn away, to deny their enduring hold on us. When Kristeva (1982, 4) pointed to the corpse – ‘that most sickening of wastes’ – as the epitome of abjection she raised the issue that has since become more familiar through the work of Derrida: that the status of the dead may be that of the spectre who refuses to be silenced and invisibilised. The abject is always something inbetween, neither wholly self nor wholly other, something that cannot be shaken off. Even as bodies degrade and dematerialise, they are still matter and they still matter. What I mean is that in their radical undecidability hovering between absence and presence, past and present, life and death, the non/living continue to accompany, and sometimes traumatise us. Any engagement with death and mourning speaks at very least to a sense of communality that unsettles singular identities and modes of representation. The non/living, the spectral traces, are never dead and gone but persist as dynamic agents in the constitution of a living present. Such dead occupy an inbetween space of affecting the living and yet seemingly offer no possibility of mutual interaction, at least in modernist thought.^11^ Nonetheless, what is demanded is an ethical response that acknowledges their irreducible enmeshment with ourselves. In Karen Barad’s words: ‘our debt to those who are already dead and those not yet born cannot be disentangled from who we are’ (2010, 264).^12^

Early in 2018 when public concern had been fully triggered, the *Mother and Baby Homes Commission* appealed for ‘personal knowledge, documentation or other information concerning the burial arrangements and/or burial places of children who died in Bessborough between 1922 and 1998' (RTÉ 2018), it again implied that the issue is one of getting the facts right, of putting everything in place and imposing order, but that is to miss the point. It speaks primarily to the epistemic limits imposed by modernism, not to any deconstruction of them. Might the task be instead to find a way forward by moving beyond moral indignation and righteous anger to fashion a different mode of living. In this respect Elizabeth Grosz offers an important reminder when she writes: ‘History is not the recovery of the truth of bodies or lives in the past; it is the engendering of new kinds of bodies and new kinds of lives’ (2003, 23). It is not that the immediate passions aroused by the uncared for dead are misplaced or misconstrued, but that they cannot answer to the continuing spectral presence of those who demand an impossible justice. Like the monster, the spectre always returns, not as an external manifestation, but as an internal companion. We *must* live well with the dead if we are to live well at all. With such reflections in mind, it is fruitful to turn again to Derrida whose thoughts on living well with the dead have inspired from the start the Irish project on which much of my empirical research is based.^13^

The very first implication of the phrase ‘living well with the dead’ is, of course, that the dead are indeed the non/living, the absent presence of others who require an ethical response. In *Spectres of Marx* (1994), Derrida developed one of his most resonant themes: that we are haunted by traces of otherness that disturb the temporal and ontological heart of being. It suggests the need for an ethics – a hauntological ethics – that takes seriously the interpolation of absent presences: those who are yet to be born, those who are no longer and even those who may never be (Henriksen 2016). It concerns not an immediate moment, but the will-have-been of the future and undecidable past. The pun of hauntology, which of course intends to bring to mind ontology, is a playful yet critically acute concept which Derrida enlists to in order to sketch out an ethics already indicated in his work on hospitality. In the context of hauntology, there is nothing that links to rules or principles, or to the concerns of conventional morality that rely on what has *already* been determined. Rather, spectral ethics sets out a desire to stay ‘with ghosts, in the upkeep, the conversation, the company, or the companionship, in the commerce without commerce of ghosts. To live otherwise, and better. No, not better, but more justly. But with them’ (1994, xviii). Unlike so many of the contemporary responses to the re-cognition of the putatively historic abuses perpetrated in Mother and Baby Homes, the Derridean approach offers no encouragement to identify the revenants and then disperse them. Instead as Henriksen puts it in her commentary on hauntology: ‘The ethical task is to stay with the uncertainty of not knowing what this something is prior to its arrival. Only in this not-knowing can the truly different and other take place’ (2016, 20).

For Derrida, the willing embrace of undecidability is at the heart of living well for it is there that modernist structures of ontology and temporality can be opened on to different horizons of living. The conventional themes of recognition and specification sound properly respectful, but they are rooted in the here-and-now that circumvents transformation by domesticating the strange or out of order. Giving an identity intends to pin down and settle uncertainty but it is more for our own sake, rather than for theirs. That is not to say that naming plays no part in Derridean ethics. Although he roundly rejects the call for identity papers (Derrida 1999a), that move does not preclude the act of embracing the other’s name without judgment and as an act of welcome. Remaining open to the arrival of otherness, here as a spectral disturbance of the proper, is what I have characterised elsewhere as an ethics of risk (Shildrick 2002). Because it is not predictable, and on arrival persists in a certain undecidability, the encounter with the other who resists being assigned a name or category can never be comfortable or assimilative.

In his use of the term ‘hospitality’, Derrida (1999b, 2000) makes clear that to welcome the unknown without reservation, whatever the potential risks, is at the heart of a non-formalised and postmodernist ethics. Where conditional hospitality usually takes the form of invitation – perhaps even to an unknown stranger – it nevertheless carries an implicit demand that certain procedures are adhered to: Come and stay in my home, but please clean the shower after use; let me hear your story so long as I can assign a name to you. This is conditional hospitality, the everyday negotiation that stays within prescribed boundaries and causes little disquiet. So long as the rules are respected on both sides, the moral requirements can be properly observed without fundamentally displacing the normative relationship between the putative self and putative other. In contrast, absolute hospitality – which like justice is finally impossible – aspires to welcome the other without preconditions or expectations. And it is precisely because the spectral other is a figure of ambiguity and difference that the welcome speaks to an ethical response. If we cannot fall back on what is already known or can be grasped by knowledge, then the moment of decision, of responsibility, is ours alone. In failing to be brought under a category, the spectral other is deeply disruptive of spatial and temporal locatedness, yet offers the chance of a different – and more just – future.

The spectre, in any case, is never simply a signifier of otherness, but an altogether more complex figure that calls to mind not so much the other per se, but the trace of the other in the self. When the host invites in the non/living, the threshold that is crossed is the very boundary between self and other. I am no longer myself but irrevocably changed by the exposure to the *arrivant*. Derrida finds himself unable to accept the finality of ‘absolute mortality (that is, without salvation. resurrection, or redemption) – neither for oneself nor for the other’ (2007, 24), and he precisely links the idea of living on – and he means for both oneself and the quasi-dead – to the spectral (26). It is then about an ethics of ‘learning to live finally’, a phrase that evokes both the arrival at a moment of beginning and the reaching of an end. Yet this is no temporal progression from birth to death along the lines of a familiar teleological narrative. Queer theory is well rehearsed in the rejection of the chrononormativity of the life course from the moment of birth to the advent of death, and of the reproductive futurity that portends (Freeman 2010; Edelman 2004),^14^ but this is something different. Here beginning and end are simultaneous and irreducible to discrete components of ‘a life’; it is not about the resistance of queer identities but about queering time. As Henriksen puts it, following Derrida, ‘learning to live (finally) is non-linear, as all haunting is, holding within itself life and death, not least through the act of mourning the loved one who is yet to die’ (2016, 143).

The evocation of mourning takes on an intriguing sense when it comes to the other within. For Derrida (1994) healthy mourning is not a mode that could gradually fade away and eventually result in closure but one that entails incorporation of the ‘lost’ other, albeit without the violence of assimilation. One must both live well with the dead and offer them a place within, and it is this very *interiority* of engagement that enables the host’s entry into the atemporal space of relationship to what has been, and what will have been. Hospitality is no longer the generosity of the self to the other, but a mutual interaction. As Derrida puts it: ‘the guest becomes the host’s host’ (2000). It is indeed what a hauntological ethics demands and a form of mourning that exceeds individual affect and cognition. In a similar but somewhat more materially situated way, Butler too sees mourning as a way of fulfilling the communality of self and other, and loss as a mode that may change my positionality for ever: ‘When we [mourn], something about who we are is revealed, something that delineates the ties we have to others, that shows us that these constitute what we are, ties or bonds that compose us’ (2004, 22). More overtly than Derrida who is primarily concerned with what is at stake for the self, Butler’s approach is firmly directed towards a collective responsibility in the face of empirical and ontological vulnerability. For her mourning is a political act as much as a personal one, and she insists that responding to the ungrieved is ‘an insurrection at the level of the ontological, a critical opening up of the questions … Whose lives are real? How might reality be remade?’ (2004, 33). But whether we call it mourning or hospitality, it is clear that the open encounter with the spectral dead is transformative.

The ambiguities of Derrida’s approach will not appeal to those seeking resolution now, but we might say that it is precisely *because* the spectral other is a figure of ambiguity and difference that hospitality invokes an ethical response that far exceeds the moral demand to name and commemorate the dead. As the many acts of remembrance held across Europe in 2018 for both the named and unnamed military dead of the first world war show, such shared mourning can generate important evocations of community, but they rarely necessitate questioning of the ostensibly fixed parameters life and death, nor cause any disturbance to the status of the self. In contrast, when the unknown resists domestication or being brought under a category, then the ethical moment of response and responsibility cannot be fulfilled by ritual and regulation alone. The spectral other, of whom Derrida speaks, is always unexpected and inherently disruptive to ontological, spatial and temporal certainties, but it signals, nonetheless the possibility of the *avenir*, the future yet to come that holds the tentative promise of a greater justice. Our responsibility in the Derridean mode is to hold open the channels of disjointed temporality, to welcome the *arrivant* who cannot be identified, or even materialised, and yet to let them speak, ‘even if it is in oneself, in the other, in the other in oneself’ (1994, 176). Instead of turning to the law for redress, or to a religious hierarchy for a belated blessing and words of comfort, could our encounter with the revenants of Tuam and Bessborough – and indeed all the others whose collective deaths have not been marked – open on to justice in an entirely different sense that accepts uncertainty and reaches beyond the present moment? The queer temporality and spatiality of coincident life/death or of absence/presence are not things to overcome; rather they are constitutive of a transformed imaginary.

As long as new revelations continue to come to light, the scandal of the Mother and Baby Homes remains firmly in the public eye, and just as Derrida warned the politics of memory entail the intervention of the state. Activists groups, feminist and otherwise, find that the issues they are addressing are all too easy reduced to a supposedly universal call for public memorialisation. At the request of the *Collaborative Forum of Former Residents*, which is assumed to speak for all, the State has in December 2019 set up a *Grant Scheme for Commemorative Events.* The intention may be benign but it seeks to effect the kind of closure that Derrida and Butler alike resist. Although forms of restorative justice seem a worthy aim, at root they are inadequate, even meaningless; what has happened cannot be undone. Yet there are practical alternatives, as the Bonafini group of the Madres de Plaza de Mayo have demonstrated.^15^ In response to the Argentinian State promise to exhume, identify and memorialise The Disappeared, those mothers opposed the scheme on the grounds that ‘however painful, the wounds of the disappearances had to be kept open to resist a national forgetting’ (Robben 2005, 144). Their preference for staying with the loss and enacting collective rather than individual motherhood for the undifferentiated remains echoes both Derrida and Butler.

Derrida’s work on spectrality and his hauntological ethics undoubtedly illuminates the discomfort that many feel in the face of revenants or more prosaically, the recovered dead. His critique gestures strongly towards a different kind of response, but I want to conclude by very briefly turning to Deleuze who more fully sets out the grounds for what might queer the psycho-cultural imaginary. If, as seems anthropologically certain, the dead have haunted the imaginaries of every age and culture and have remained active agents in the lives of the living (Laqueur 2016), then perhaps the category of death itself should invite further exploration. Where Derrida rejects absolute mortality and speaks of living on (finally), he implicitly gestures towards the kind of interconnections that Deleuze and Guattari (1987) call assemblages. In the further Deleuzian mode, dying is both a personal event, experienced within a conventional time frame that gives rise to the desire to memorialise what is lost, and also a continuing atemporal process that exceeds the binary of life/death. The threshold of mortality marks the cessation of individual being but is displaced by the continuity of becoming together.

In the wider perspective, the decisive break at the heart of Deleuze’s philosophy is his contestation of the notion of the singular subject of modernity – in brief, the static state of ‘being’ marked by the capacity to say I am. Instead in Deleuzian thought any subject is always in a process of unravelling, not as a negative disintegration but as the opening to the positivity of a state of becoming. Each of us is caught up in assemblages, the multiple and unpredictable webs of connections with material and non-material others, in which life itself is characterised as a vitalist force that exceeds the unique interests and experiences of each individual (see Braidotti 2006). For Deleuze, then, life is marked by potentiality, by the generative power of connection and the processes of transformation. In short, life is not a discrete essence bookended at the beginning and end by non-existence. Although Deleuze acknowledges that life in the everyday sense is continually actualised and embodied in the individual where it has *personal* value, his point is that that body is simply an element in the broader cycle of becoming. As such, the death of a human being is in an important sense the final dissolution of a singular existence, yet not an absolute closure. Rather it is the point at which the materialisation of individual life is subsumed by a non-personal vitalism. Organisms may die, but life itself endures. Certainly, *any* death marks the limit of certain personal relations and projects and the cessation of a particular self, but the temporal event of dying is a further opening, another moment of becoming. As Deleuze understands it: Death has an extreme and definite relation to me and my body and is grounded in me, but it also has no relation to me at all – it is incorporeal and infinitive, impersonal, grounded only in itself. (1990, 151)

In the liberal humanist context in which only individual rights and the hope of personal justice count, it is understandable that the re/covery of the unnamed dead should arouse passionate feeling, but nonetheless we need to turn to another register. In the Deleuzian rethinking of mortality, such personalised affect and expectation gives way to the intensity of continued becoming in a process that does not meet a limit in death. In that sense, the multiple unrecorded deaths of uncared-for neonates and infants are a legal, social, and often familial, scandal, but they continue to contribute to the ongoing flux and flow of life, not least through the spectral absence/presence of which Derrida speaks. What matters in Deleuzian terms is neither guilt nor anger about the infants’ untimely demise nor any obligation to bear witness to them, but the capacity to affirm life, both in its renewed potential and in its endurance. A successful outcome to the sociopolitical scenario I have outlined would not be about establishing the grounds for a guilt-free reburial and forgetting, but about what Braidotti has called ‘sustainability’ (2006, 129), ‘the very possibility of the future, of duration, of continuity’ (2006, 137), by which she means the desire to enhance one’s possibilities through pleasure and pain alike.^16^ It is about the capacity to overflow individual boundaries – including those of life and death – and about transformation even in the face of adversity, and the move towards new possibilities of becoming other. If the event of dying were seen as the recomposition of life under new relations of communality, then mortality itself would not be an abject failure – grievable or otherwise – but rather the opening to new and productive interconnections.

For the time being, our sociocultural imaginary is caught up in modernist conceptions of time, of life and death, absence and presence, and the separation of self and other, that generate deep and seemingly unavoidable anxieties when the dead refuse to stay silent. The events in Ireland and elsewhere inevitably provoke the demand for an immediate political response, but they also suggest new ways of thinking existence, not in terms of a singular time span, but through dynamic coexistence and forms of assemblage that blur the boundaries between life and death. If we are to effect change in the psychocultural imaginary, then one way would be to openly explore what lies beyond the immediate wound of the event of death by rethinking our investments in the singular self and in chrononormativity. We should take comfort that there are already queer ways of thinking death that link to a wider atemporal and impersonal vitalism. It does not diminish the immediate pain or grief but opens onto another dimension where as Braidotti puts it, death – rather than being disturbed and disturbing – frees us into life.

## Notes

1 A comparatively dispassionate UN document reported: ‘For some children … particularly in Canada, Australia, and the United States, boarding school experiences are particularly brutal. Thousands of children did not survive these schools, either through neglect, inadequate medical care, inadequate food, or even in some cases, murder and torture’ (Smith 2009, 29).

2 Thanks to Radomska (2016) for developing the usage.

3 In the later years the Centre, which operated between 1876 and 2009, increasingly housed adult residents. As well as high numbers of unexplained deaths, the Centre was notorious for its neglect and abuse of residents. In 2013 following court action, survivors were awarded compensation and the Government of Ontario offered a formal apology.

4 An RTÉ television serialisation of *The Handmaid’s Tale* was coincident with the emergence of the Tuam scandal.

5 The Roman Catholic concept of Limbo, though never official doctrine, was effectively repealed in 2007.

6 The adoption scenario remains somewhat speculative, and an alternative explanation for the absence of marked graves has emerged from an interim report from the official Commission (April 2019). It appears that a significant number of infant bodies from a similar Home in Dublin were passed on to anatomy labs, and the same may be true of Bessborough.

7 Wilmer (2015), citing Martin Sixsmith, claims that because infants born in Mother and Baby Homes were denied medical care during birth and subsequently prevented from breast feeding, many failed to thrive and were malnourished, and that up to 68% of them died.

8 A 5th Interim Report was issued by the Commission in April 2019, but the inquiry remains ongoing.

9 See the wrenching account written by feminist scholar O’Donnell (2018) of her research into Magdalene institutions.

10 See Shildrick (2019) for more on my understanding of temporality.

11 See Langford (2013) for the persistence of a very different southeast Asian understanding. In contrast, Nils Bubandt claims that spirits in both the East and the West ‘are becoming implicated in the globalisation of an interiorised and psychologised understanding of what it means to be human’ (2012, 1).

12 Tadeuz Kantor’s performance piece *The Dead Class* is a stunning exemplification of the potentially hostile relation of the unacknowledged dead within.

13 The project *Living Well with the Dead* is situated at University of Cork and funded by the Wellcome Trust [grant no. R18190]. I acknowledge my very productive collaboration with Orla O’Donovan, Joan McCarthy and Roisin O’Gorman, and the many others who have attended our workshops.

14 I do not mean to imply that either Freeman or Edelman are limited to this narrow reading.

15 I am grateful to the anonymous reviewer who drew my attention to this.

16 It is noticeable that in a 2018 interview, a *Remember Every Name* activist said that a gathering at the burial site was not just about memorialising the dead but a desire to ‘honour the strength and resilience of the survivors’.
